# What GDPR and the Health Research Regulations (HRRs) mean for Ireland: “explicit consent”—a legal analysis

**DOI:** 10.1007/s11845-020-02331-2

**Published:** 2020-07-30

**Authors:** Mary Kirwan, Blanaid Mee, Niamh Clarke, Aoife Tanaka, Lino Manaloto, Emma Halpin, Una Gibbons, Ann Cullen, Sarah McGarrigle, Elisabeth M. Connolly, Kathleen Bennett, Eoin Gaffney, Ciaran Flanagan, Laura Tier, Richard Flavin, Noel G. McElvaney

**Affiliations:** 1 Department of General Practice, Royal College of Surgeons in Ireland, Dublin, Ireland; 2 Department of Histopathology, St James’s Hospital, Dublin, Ireland; 3 Department of Surgery, School of Medicine, Faculty of Health Sciences, Trinity College Dublin, Dublin, Ireland; 4 Biobank Ireland Trust, Dublin, Ireland; 5 School of Biotechnology, Faculty of Science and Health, Dublin City University, Dublin, Ireland; 6 Conway Institute of Biomolecular & Biomedical Research, University College Dublin, Dublin, Ireland; 7 Department of Surgery, St James’s Hospital, Dublin, Ireland; 8 Department of Epidemiology and Public Health Medicine, Royal College of Surgeons in Ireland, Dublin, Ireland; 9 Department of Medicine, Royal College of Surgeons in Ireland, Dublin, Ireland

**Keywords:** Common law, Consent declaration, Explicit consent, GDPR, Health research, Informed consent, Ireland, Irish health regulations, Research

## Abstract

**Background:**

Irish Health Research Regulations (HRRs) were introduced following the commencement of the General Data Protection Regulation (GDPR) in 2018. The HRRs set out supplementary regulatory requirements for research. A legal analysis presented under the auspices of the Irish Academy of Medical Sciences (IAMS) on April 8 and November 25, 2019 at the Royal College of Surgeons in Ireland welcomed the introduction of GDPR and the HRRs. The analysis found the GDPR “explicit consent” introduced by the HRRs is problematic. A call was made to regulate informed consent in line with the common law as an achievable alternative safeguard, bringing Ireland in line with other EU Member States.

**Aims:**

This article aims to review academic papers, legal opinion, EU opinion and advice and data protection law in relation to research and explicit consent, in order to examine the legal burden of GDPR and the HRRs on health research in Ireland and to determine whether the analysis presented at the IAMS meetings is reflected more widely in legal text.

**Methods:**

Legal literature review of academic papers, legal opinion, EU opinion and advice and data protection legislation.

**Results:**

The legal literature review overwhelmingly supports the concerns raised.

**Conclusions:**

Our results confirm the GDPR explicit consent requirement of the HRRs is having had a significantly negative and far-reaching impact on the conduct of health research in Ireland. Urgent review of the HRRs and meaningful engagement between the health research community and legislators in healthcare is required.

## Introduction

The European Union (EU) General Data Protection Regulation (GDPR) came into effect on May 25, 2018. Article 9 (4) of GDPR permits Member States to introduce further protections or safeguards, as regards personal data, including health data [1–3]. Ireland’s Health Research Regulations (HRRs) followed on August 8, 2018 introducing additional regulatory requirements for health research in relation to governance, processes and procedures have impacted on several aspects of research [4, 5]. One of the requirements of the HRRs is that identified or identifiable personal data cannot be included in health research unless (a) GDPR “explicit consent” exists or (b) a consent declaration has been granted [1–7].

At public meetings organised by the Irish Academy of Medical Sciences (IAMS), on April 8 and November 25, 2019 at the Royal College of Surgeons in Ireland, GDPR and the HRRs were warmly welcomed. It was acknowledged that informed consent is central to the fundamental rights of research participants and always has been at the core of health research. Yet the view was expressed that the additional mandatory GDPR explicit consent requirement imposed by the HRRs is a significant impediment to conducting research in Ireland. A call was made to regulate informed consent in line with the common law as an achievable alternative safeguard in the Irish health research setting and would align Ireland with the approach taken in other EU Member States [8].

It was felt that the difficulties identified warranted further investigation. This paper sets out to examine the impact of GDPR explicit consent and the HRRs by way of literature review of academic papers, legal opinion, EU opinion, and advice and data protection law.

## Methods

### Legal literature review

A comprehensive desk-based literature review of relevant legislation, regulation, academic literature, legal opinion and various European bodies and institutions opinions in order to frame the legal and regulatory challenges for Irish researchers [9–19].

## Results & discussion

### Introduction to explicit consent, GDPR and the HRRs

The Data Protection Act 2018 gives national effect to aspects of GDPR that are specific to Ireland [17]. In exercise of the provision for suitable and specific measures for processing data set out in Section 36 (2) of the Data Protection Act 2018, the Minister for Health signed the HRRs into effect in August 2018 thus establishing a number of conditions for the processing of personal data for health research purposes. Of these safeguards, explicit consent has emerged as a particular challenge for researchers. These challenges are outlined in the companion paper: What GDPR and the Health Research Regulations (HRRs) mean for Ireland: a research perspective.

GDPR recognises the importance of science and innovation and is not designed to impede research but rather to facilitate the free flow of information. To that end, it affords scientific research a privileged position within the Regulation, carving out a research exemption for processing special categories of personal data at Article 9 (2)(j). While explicit consent is another lawful basis of processing under Article 9, it is not a mandatory requirement. The HRRs in effect negate the research exemption by applying a mandatory GDPR explicit consent safeguard to the processing of personal data for the purpose of health research.

In saying that, the use of personal data without explicit consent is permitted in exceptional circumstances. To avail of this concession, researchers must submit an application to the national Health Research Consent Declaration Committee (HRCDC), established by the HRRs, for a consent declaration, demonstrating that substantial public interest exists and that GDPR explicit consent is not feasible among other requirements.

### Informed consent and GDPR explicit consent—what is the difference?

#### Informed consent

Informed consent has long existed as an ethical and legal requirement for the conduct of research in Ireland [9, 10].

The HSE (Health Service Executive) National Consent Policy [10] sets out the requirement of informed consent when conducting research. The policy states that in advance of carrying out research, consent documentation containing all information necessary to make an informed decision is submitted to a research ethics committee (REC) for consideration as to whether it is adequate to achieve consent.

The policy also recognises that informed consent is not always possible in circumstances such as adults lacking decision-making capacity, emergency situations, epidemiological research, public health emergencies, archival material and research involving deceased persons. It specifies that a REC may waive informed consent subject to conditions.

#### GDPR explicit consent

GDPR consent is one of the six lawful bases to process personal data listed in Article 6 of GDPR.

Explicit consent is one of ten Article 9 bases which allow for the processing of special categories of personal data, such as health data. The term explicit refers to the way in which the GDPR consent is expressed by the data subject and raises the standard of the consent where there is a serious data protection risk.

The concept of consent in the Data Protective Directive (Directive 95/46/EC) has evolved, and GDPR sets out stricter requirements for obtaining valid consent from data subjects [19]. In practice, GDPR raises the bar as regards implementing consent [11] with validity relying on cumulative criteria set out in Article 4 (11), Article 7 and recitals 32, 33, 42, and 43 of GDPR being met.

#### What is the difference?

The difference between the two types of consent lies in purpose and conditions. Informed consent preserves privacy of the person, autonomy, bodily integrity and ethical standards. GDPR explicit consent relates to data processing and is one of the mechanisms set out in GDPR to protect informational privacy. Importantly, the conditions necessary to achieve validity differs between the two. GDPR explicit consent sets out more criteria and conditions than informed consent; see Table 1.

**Table 1:** Not all consent is the same

Informed consent [7]	GDPR consent [1]	GDPR explicit consent [1]
• Have received sufficient information in a comprehensible manner about the nature, purpose, benefits and risks of an intervention/service or research project	• Freely given—no imbalance of power, not conditional, granular, without detriment	•The term “explicit consent” simply refers to the way GDPR consent is expressed by the data subject. It means that the data subject must give an express statement of consent. An obvious way to make sure consent is explicit would be to expressly confirm consent in a written statement.
• Not be acting under duress	• Specific	
• Have capacity to make the particular decision	• Informed	
	• Unambiguous	
	• Unbundled	
	• Active opt-in	
	• Documented	
	• Easy to withdraw	
	• No blanket A valid GDPR consent depends on these cumulative criteria being present.	

Recent opinion from the European Data Protection Supervisor (EDPS) issued in January 2020 recognised the difference between the two types of consent and favours of the use of “informed consent” as a safeguard of processing and not GDPR explicit consent:

It states: “There may be circumstances in which consent is not the most suitable legal basis for data processing, and other lawful grounds under both Articles 6 and 9 GDPR should be considered. However, even where consent is not appropriate as a legal basis under GDPR, informed consent as a human research participant could still serve as an ‘appropriate safeguard’ of the rights of the data subject. Under what conditions such informed consent might be deemed an appropriate safeguard is still unclear” [14].

This opinion mirrors the call for the regulation of informed consent for research as an alternative safeguard  to “GDPR explicit consent” presented by IAMS at public meetings, on April 8 and November 25 2019, at the Royal College of Surgeons, Ireland [8].

### The HRRs and the explicit consent requirement

The HRRs’ mandatory GDPR explicit consent requirement adds an additional layer of consent, on top of the pre-existing legal and ethical requirement of informed consent. In practice, this means when conducting health research,  a researcher must have an Article 6&9 lawful basis as well as the GDPR explicit consent  safeguard and informed consent, arguably placing an unnecessary burden of double consent on patients.

The requirement is at odds with emerging opinion from various European bodies and institutions that have stated GDPR consent will not always be appropriate for health research, particularly in relation to clinical trials [11–14].

Legal commentators have noted: “Organisations involved in clinical trials and health research in Ireland are now faced with a European Data Protection Board (EDPB) opinion that conflicts with Irish legislative requirements…. It may be disconcerting to such organisations to be required to take steps in Ireland that seem contrary to the EDPB’s view of best practice and are likely to be divergent with the approaches taken in other Member States” [12].

Examples of other Member States taking a divergent approach include the UK, Sweden and France where consent for research is not explicit consent as defined in GDPR and but is another type of consent legislated for separately [20, 21]. Denmark has taken full advantage of the research exemption set out in Article 9 (2) (j) and Article 89 of GDPR under Section 10 of the Danish Data Protection Act 2018 and Section 46.1 Health Act.

### Problem areas

The HRRs create multiple problems for Irish researchers:

1. The achievability of the criteria for valid consent particularly in relation to the “freely given” requirement where there is the existence of a “power imbalance”
2. The technical and bureaucratic burden
3. Blanket application of GDPR explicit consent

### Achievability

The validity of consent relies on cumulative criteria set out in Article 4 (11), Article 7 and recitals 32, 33, 42, and 43 of GDPR being met. There is little doubt that achieving these requirements is burdensome and difficult, if not often impossible in a health research setting particularly with the existence of the power imbalance.

The EDPB, in an opinion on the interplay between the Clinical Trials Regulation (CTR) and the General Data Protection Regulation, states that in order to comply with GDPR, consent must be freely given without the existence of a power imbalance [12].

It provides the following as an example of power imbalance where consent is not considered freely given:

“….. when a participant is not in good health conditions, when participants belong to an economically or socially disadvantaged group or in any situation of institutional or hierarchical dependency” [12].

This example relates to most health research situations creating difficulty for researchers, as European opinion clearly states GDPR consent is not always appropriate but the HRRs make GDPR explicit consent mandatory. Breach of the requirements carries the potential of litigation, hefty fines and sanction in line with Articles 77–84.

### Technical and bureaucratic burden

Coming to the second problem, Donnelly and McDonagh speak to the bureaucratic burdens: “……the procedures and requirements which the HRRs require are likely to quell the enthusiasm of even the most enthusiastic researcher. Every research project in which a consent exemption is sought (no matter how small) will require REC approval; a DPIA (Data Protection Impact Assessment); the appointment of a data protection officer and compliance with substantial procedural requirements. Taken together, these requirements are likely to have a chilling effect on health research, especially in respect of participants for whom personal consent is not an option” [16].

These burdens are also reflected in the companion paper: What GDPR and the Health Research Regulations (HRRs) mean for Ireland: a research perspective.

### Blanket application of GDPR explicit consent

The HRRs’ undifferentiated blanket application of GDPR explicit consent has created significant problems in the areas of retrospective cart reviews, pre-screening, emergency research, capacity, bio-banks and the need to re-consent previously obtained consent in order to achieve the new legal standards of GDPR explicit consent.

In relation to re-consent, the Working Party 29 Guidelines on Consent state: “If a controller finds that the consent previously obtained under the old legislation will not meet the standard of GDPR consent, then controllers must undertake action to comply with these standards, for example by refreshing consent in a GDPR compliant way” [11].

This requirement created the necessity for researchers nationally to review previously obtained consents and refresh where necessary. This raised serious ethical concerns about re-contacting patients. The difficulties presented by this task have been comprehensively set out in the companion paper What GDPR and the Health Research Regulations (HRRs) mean for Ireland: a research perspective.

#### National and international academic opinion

Academic opinion on the HRRs and the mandatory explicit consent requirement has been negative.

Dove and Chen in their analysis do not support the model espoused by Ireland’s Health Research Regulations 2018, and feel consent is privileged to too great an extent [17]. They state:

“We share the concern from many in the research community that by mandating explicit consent, subject only to a committee waiver whereby it is demonstrated that (among other things) the public interest in carrying out the research ‘significantly outweighs’ the public interest in requiring the explicit consent of the data subject, many health research projects will be subject to disproportionate, burdensome regulation that will dampen health research activity in the country. This will come at a cost to research competitiveness and patient access to innovative diagnostics, drugs, and devices” [17].

Donnelly and McDonagh similarly note the damaging effect on Irish research “……. for a jurisdiction, like Ireland, which adopt a demanding (and undifferentiated) approach to the consent requirement, the consequences may well be exclusion from European-wide health research projects and an overall reduction in research projects involving research participants/data subjects who are unable to provide personal consent” [16].

While Clarke states: “The Department of Health has taken a unique and arguably restrictive approach to data protection in Ireland which is quite at variance from our European colleagues and…… will impact negatively on patient care and clinical research in Ireland”. In particular the paper outlines the problems around the burden of re-consent, retrospective chart review, capacity, pre-screening and bio-bank/archival material, due to an overly simplistic blanket application of GDPR explicit consent [3]. Promised amendments attempting to rectify some of these areas are still pending, and there is no legislative support for some current concessions in relation to retrospective chart review [8].

A survey of Non-Consultant Hospital Doctors (NCHD’s) reflects on the ground the concerns expressed by many [18]:

- 82% felt there will be new barriers and challenges to conducting research and sharing data with international colleagues
- 77% felt there would be increased time and cost commitments when conducting research
- 93% felt that GDPR and the HRRs would have implications on research
- 98% believing that health research would be more challenging
- 80% reported that patient recruitment would become more challenging
- 95% felt the regulations will lead to a selection bias in future recruitment and participation in research
- 86% felt patient who lack capacity to consent will be excluded from participating in research
- Only 23% of NCHDs felt that patients would benefit from improved privacy as the aim of the HRRs has been lost amidst worry and focus on potential infringements

Worryingly, it was felt the prohibitive research environment in Ireland may result in the loss of junior doctors to pursue research in other jurisdictions, where research is more easily undertaken [18].

#### US example

The Health Insurance Portability and Accountability Act (HIPAA) Privacy Rule was implemented by the US Department of Health and Human Services (HHS) in April 2003. A common law jurisdiction like Ireland, it similarly created a prescriptive data protection “consent” requirement for research in addition to the to the pre-existing necessity for informed consent under the Common Rule. The Privacy Rule “Authorization” resembles GDPR explicit consent, though is not as burdensome. It is also highly punitive in the event of non-compliance. A number of bodies investigated the impact of the Privacy Rule on research. Those results closely echo the current Irish experience as set out by the companion paper What GDPR and the Health Research Regulations (HRRs) mean for Ireland; a research perspective.

Findings from the Association of American Medical Colleges concluded that the Privacy Rule [22] (1) reduced patient recruitment, (2) increased the likelihood of selection bias, (3) increased the costs of conducting research by requiring more paperwork and complicating the Institutional Review Board (IRB) approval process, (4) increased the number of errors in research when de-identified information was used, (5) made multisite trials more difficult because of variations in IRB interpretation of the rule, and (6) caused researchers to abandon projects because of the increased number of rules for operating a research study.

A National Cancer Advisory Board survey of health researchers reported that (1) the Privacy Rule increased patient confusion, (2) the Privacy Rule’s complex documentation requirements delayed research, (3) differing interpretations of the Privacy Rule made conducting health research more challenging, and (4) the Privacy Rule created new barriers to the use of patient specimens collected during clinical trials [23].

Lastly, a National Survey of Epidemiologists found that only 11% of respondents indicated that the Privacy Rule strengthened public trust in research [24].

The Privacy Rule created selection bias with fewer patients agreeing to participate in research since its implementation. The complicated and lengthy authorization forms, required by the Privacy Rule, also proved an impediment to recruitment, while small healthcare entities and bodies serving disadvantaged populations were less likely to participate in research, due to an inability to meet the exhaustive Privacy Rule requirements. Thus, minority populations were underrepresented in many research studies [25].

In 2019, the Department of Health and Human Services sought consultation to revise the HIPAA Privacy Rule and identify regulatory burdens.

Some proposals have included:

- Exempting retrospective chart review from the Privacy Rule
- Aligning the Privacy Rule with Common Rule informed consent
- Permitting data disclosure without consent, where the appropriate protections exist and, in a manner similar to direct care and treatment

#### COVID-19 and the HRRs

Ireland’s GDPR explicit consent requirement remains in place at a time of national crisis with the emergence of COVID-19. This is in contrast to the UK where The Secretary of State for Health and Social Care in the UK has temporarily suspended its consent requirement under the common law duty of confidentiality in response to COVID-19 [26].

EDPB Guidelines on the processing of data concerning health for the purpose of scientific research in the context of the COVID-19 outbreak once again draw attention to the issues around the use of GDPR explicit consent: “It has to be noted that all the conditions for explicit consent must be fulfilled….. consent must be freely given, specific, informed, and unambiguous, and it must be made by way of a statement or ‘clear affirmative action’” [27].

It goes on to say: “As stated in Recital 43, consent cannot be considered freely given if there is a clear imbalance between the data subject and the controller…”

The guidelines specify the data subject should not whatsoever be in a situation of dependency with the researchers that could inappropriately influence the exercise of their free will [27]. Despite the difficulties in discharging these conditions in a health crisis, researchers here must still obtain GDPR explicit consent or submit a HRCDC application (albeit with the concession of an expedited COVID-19 review) to obtain a consent declaration where demonstrable and substantial public interest exists, and explicit consent is not feasible.

In effect, the HRRs GDPR explicit consent requirement short-circuits the pandemic exemptions set out in GDPR, and while COVID-19 studies are being undertaken nationally, it places a disproportionate burden on researchers.

## Conclusion

The findings presented at the IAMS on April 8th and November 25th 2019 at the Royal College of Surgeons, Ireland are upheld.

It is important to state that the intention of the legislators was not, in the authors’ opinion, to impede health research. Rather, GDPR presented a mechanism to regulate research and simultaneously afford greater autonomy to health research participants. Inadvertently, however, the HRRs have heavily impacted on Ireland’s capacity to conduct health research, including clinical trials (both interventional and non-interventional) and caused significant damage to Irish research.

The authors seek the regulation of consent in health research to a high, achievable standard ensuring public trust and continued support of research.

## Recommendations

1. Several proposed amendments were presented at both IAMS events in an attempt to rectify some of the problems created by the HRRs. Despite this these amendments have not been enacted, and there has been no attempt by the Department of Health to set up a consultative process with IAMS to discuss these proposals. This is an obvious area which needs to be addressed.
2. There must be an urgent review of the HRRs GDPR explicit consent requirement. 
3. A global review of the HRRs.
4. The regulation of informed consent in research is in line with the common law and the approach taken by other EU Member States.
5. Meaningful dialogue and consultation between legislators and key stakeholders including IPPOSI (Irish Platform for Patient Organizations, Science and Industry), patient advocate groups and the research community is urgently needed.

## Data Availability

Not applicable.
